# ClearSight-RS: A YOLOv5-Based Network with Dynamic Enhancement for Remote Sensing Small Target Detection

**DOI:** 10.3390/s26010117

**Published:** 2025-12-24

**Authors:** Jie Yuan, Shuyi Feng, Hao Han

**Affiliations:** 1 College of Computer Science and Technology, Nanjing University of Aeronautics and Astronautics, Nanjing 210024, China; yuanjie_6952@163.com (J.Y.); feng_shu_yi@aliyun.com (S.F.); 2 Shanghai Aerospace Electronic and Communication Equipment Research Institute, Shanghai 201109, China

**Keywords:** remote sensing image, small target detection, YOLOv5, dynamic enhancement, attention mechanism, target recognition

## Abstract

Small target detection in remote sensing images faces challenges due to complex backgrounds, weak features, and large scale differences. This paper proposes an improved YOLOv5-based network, termed ClearSight-RS, with the full name “Clear and Accurate Small-target Insight for Remote Sensing”. As the name implies, the network is dedicated to achieving clear feature perception and accurate target localization for small targets in remote sensing images. The improvements focus on three aspects: integrating an improved Dynamic Snake Convolution (DSConv) module into the backbone network to strengthen the extraction of small target boundaries and geometric features, as well as the expression of weak textures; embedding a Bi-Level Routing Attention (BRA) module in the Neck part to enhance target focusing and suppress background interference; and optimizing the detection head by retaining only shallow high-resolution feature layers for prediction, reducing feature loss and redundant computations. Experimental results show that, based on the VEDAI dataset, ClearSight-RS achieves the highest mAP for all 8 vehicle categories; based on the NWPU VHR-10 dataset, its overall mAP reaches 93.8%, significantly outperforming algorithms such as Faster RCNN and YOLOv5l; based on the DOTA dataset, the capability of the proposed BRA module in suppressing background interference and capturing small target features is demonstrated. The network balances accuracy and efficiency, performing prominently in detecting vehicles and multi-category small targets in complex backgrounds, verifying its effectiveness.

## 1. Introduction

With the rapid development of UAV and satellite remote sensing technologies, the volume of remote sensing images has exploded, providing a rich data foundation for intelligent remote sensing analysis. Small target detection in remote sensing holds significant application value in various fields such as environmental monitoring, maritime rescue, urban construction, and geological disaster monitoring [1,2]. Especially with the continuous advancement of high-resolution imaging systems, automatic detection and recognition of ground objects from remote sensing images have become a research hotspot. Among them, small target detection has attracted much attention due to its significance in precise identification of key targets like ships, vehicles, and aircraft, which is crucial for safety early warning and emergency response.

However, remote sensing images often suffer from problems such as dense targets, small target sizes, and blurred texture features, posing great challenges to small target detection. Accurately and robustly detecting these tiny but information-critical targets in complex backgrounds remains a core problem to be solved in current remote sensing target detection research.

Traditional remote sensing image target detection methods mainly rely on manual feature extraction and description, such as Histogram of Oriented Gradients (HOG) [3], Scale-Invariant Feature Transform (SIFT) [4], etc. Although these methods achieved certain results in the early stage, they generally have limitations such as limited feature expression ability, weak generalization ability, and complex design process, making it difficult to meet the diverse needs of current large-scale and high-resolution remote sensing images. With the rapid development of deep learning technology, models such as Convolutional Neural Networks (CNNs) have shown superior performance in computer vision tasks such as image segmentation and target detection, prompting more and more researchers to introduce deep learning methods into the field of remote sensing image analysis to improve detection accuracy and automation.

With the rapid development of artificial intelligence technology, the application of deep learning methods in remote sensing image target detection has become increasingly popular, and detection algorithms based on deep learning have achieved remarkable success in the field of image detection [5,6]. At present, deep learning-driven target detection algorithms are mainly divided into two categories: single-stage detection frameworks and two-stage detection frameworks. Two-stage detection algorithms first extract candidate boxes and then recognize the targets in these candidate boxes. Classic two-stage detection algorithms include the R-CNN series (such as Fast R-CNN [7], Faster R-CNN [8], and Mask R-CNN [9]). When facing scenes with dense small targets, two-stage algorithms may generate a large number of candidate boxes, leading to redundant calculations and failing to meet real-time requirements. The other category is single-stage algorithms, which omit the candidate box extraction stage and use a single network to directly predict the position and category of objects from the input image, saving a lot of computing power and significantly accelerating the detection speed. Classic single-stage algorithms include SSD [10], YOLO (You Only Look Once) series [11,12], etc. Among them, the YOLO series [13,14,15,16,17,18] are widely used due to their low computational cost and high efficiency, becoming one of the mainstream algorithms for target detection.

Beyond CNN-based architectures, Transformer-based methods represented by DETR [19] have gained attention due to their global feature modeling capability. DETR utilizes self-attention mechanisms to model target relationships, which aids in capturing scattered small targets in remote sensing images [20]. Additionally, Mamba-based backbone networks [21] have emerged as a new direction. Mamba’s ability to capture long-range dependencies with low computational cost has been explored in remote sensing object detection [22]. However, its inherent focus on sequential feature processing leads to insufficient preservation of local details—a critical aspect for small targets with weak texture features.

Practical remote sensing small target detection (e.g., real-time UAV monitoring, rapid satellite interpretation) demands both high accuracy and efficient computation for feasible deployment. Existing deep learning methods often face a trade-off: some boost accuracy via deeper networks or complex modules but suffer from excessive parameters and slow inference, while others prioritize lightweight design at the cost of weak small-target performance. Balancing accuracy, computational efficiency, and model lightweight thus becomes another key challenge, and a core goal of this study.

This paper focuses on the task of small target detection in remote sensing images and proposes structural improvement strategies for a series of outstanding problems existing in the application of existing target detection methods in remote sensing scenarios. Specifically, traditional detection models tend to have the following shortcomings when processing remote sensing images, which are the three core challenges of remote sensing small target detection: first, small targets have weak features that are easily lost during feature propagation, leading to a high missed detection rate as they are submerged in deep networks; second, remote sensing images have complex backgrounds, which easily interfere with small targets and cause confusion between foreground and background, further interfering with target positioning and classification; third, fixed convolution kernels in convolutional neural networks are difficult to adapt to irregular contours of small targets, and multi-scale detection branches have redundancy and interference, while the lack of long-range dependency modeling ability also limits the capture of global contextual semantics for small targets.

Among various convolutional methods, we select DSConv [23] as the core enhancement module for three key reasons: First, compared to traditional deformable convolutions that only adjust sampling positions without continuity constraints, DSConv’s snake-shaped sampling path can more effectively capture the irregular contours of small remote sensing targets (e.g., ships at arbitrary orientations, scattered small vehicles), which aligns well with the “weak boundary features” challenge of small targets. Second, unlike static adaptive convolutions that rely on fixed rule-based adjustments, DSConv employs learnable offsets to achieve dynamic adaptation to local background complexity, effectively suppressing background noise while preserving target details—this is crucial for remote sensing images with high background–target interference. Third, DSConv’s lightweight design does not introduce excessive parameters, outperforming heavy dynamic convolution methods (e.g., DyConv [24]) that increase computational burden. These characteristics make DSConv the optimal choice for enhancing small target feature extraction in remote sensing scenarios.

Therefore, aiming at small target detection in remote sensing images, this paper proposes an improved network based on YOLOv5, named ClearSight-RS, which focuses on enhancing the detection performance of small targets in remote sensing images through dynamic enhancement mechanisms. First, to strengthen the model’s perception of small target boundaries and geometric structures, an improved DSConv module is integrated into the backbone network. Leveraging dynamic receptive fields and deformable structures, it enhances feature extraction capabilities, improves the expression of weak textures in small targets, and maintains computational efficiency. Second, addressing the issue that small targets are easily overwhelmed by complex backgrounds in remote sensing images, a Bi-Level Routing Attention (BRA) module is embedded in the Neck part. This module reinforces the model’s attention focus on target regions and effectively suppresses redundant background interference. Third, considering that small targets in remote sensing images occupy limited image space and are prone to feature loss, the original detection head structure is optimized by retaining only the shallowest high-resolution feature layer for prediction. This modification improves the perception accuracy of tiny targets while reducing redundant computations. Finally, systematic evaluations of the proposed ClearSight-RS on multiple remote sensing small target detection datasets demonstrate that it achieves a favorable balance between accuracy and efficiency, with particularly superior performance in dense small target areas. The code of ClearSight-RS is available at https://github.com/cocotorrow/ClearSight-RS.git (accessed on 6 December 2025).

## 2. Related Work

### 2.1. Research Task Definition

Remote sensing small target detection aims to accurately identify and locate objects with small pixel scales in aerial/satellite images, such as small vehicles, ships, and infrastructure. Unlike general object detection, this task faces three unique challenges: (1) Weak feature expression: Small targets have limited texture and shape information, leading to insufficient feature discrimination; (2) Complex background interference: Remote sensing images contain a large number of redundant backgrounds (e.g., vegetation, water bodies, clouds), which easily overwhelm weak target features; (3) Irregular target distribution: Small targets are often scattered and have arbitrary orientations, increasing the difficulty of feature aggregation.This study focuses on the bounding box detection task of small targets in remote sensing images, with the goal of improving detection accuracy while maintaining lightweight and real-time performance. The research scope covers three mainstream datasets (VEDAI, NWPU VHR-10, DOTA) to verify the generalization of the proposed method.

### 2.2. Related Improved Algorithms

To address the prevalent issue of complex backgrounds in remote sensing images, relevant studies have carried out targeted improvement. Su et al. [25] constructed a scale-stratified feature pyramid structure and proposed a center regression strategy fused with distance constraints, effectively improving the model’s adaptability to remote sensing target detection in complex scenarios. Liu et al. [26] focused on the darknet residual blocks of YOLOv3, strengthening the preservation of spatial information by introducing convolution operations in the shallow layers of the network. Zhu et al. [27], based on YOLOv5, replaced the original prediction head with a self-attention module (Transformer), weakening the interference of complex backgrounds and improving the detection performance of remote sensing targets. Xiao et al. [28] proposed a feature decoupling module using an attention mechanism and a localization refinement network that automatically optimizes anchor box parameters to achieve more accurate localization.

To solve the problem of blurred remote sensing targets, detection based on regional context information can be adopted, which combines information beyond the target and the global information of the image to enhance feature representation, thereby alleviating the issue of blurred target appearance to a certain extent. In 2019, Ma et al. [29] designed a context information fusion sub-network that integrates local contextual features and target-to-target relational contextual features to handle the complexity of target object appearance. In the same year, the CAD-Net proposed by Zhang et al. [30] enhances the feature expression of targets by learning global and local contextual features of targets, while introducing an attention mechanism to focus on target features in the detection region. Chen et al. [31] extracted region-of-interest feature maps based on FPN, then fused these extracted feature maps with the feature map of the entire image to obtain contextual information for detection.

Remote sensing images contain a large number of small targets with weak features. To tackle this problem, researchers have explored various solutions such as data augmentation, super-resolution reconstruction, and multi-scale learning. In 2018, Zhang et al. [32], based on Faster RCNN, added a deconvolution layer after the last convolutional layer of the base network for small target detection; in the same year, Yang et al. [33] proposed a new detection model based on a multi-task rotated region convolutional neural network, which strengthened the feature expression ability of small targets through a densely connected structure.

In recent studies, YOLOv5 has become a hot optimization topic due to its performance advantages. Luo et al. [34] improved the feature extraction module of the YOLOv5 backbone network, and verified the improvement in detection performance in large-scale dataset validation. Zhang et al. [35] further upgraded the model by not only improving the pyramid pooling module and embedding an attention mechanism but also introducing a Bi-FPN structure to enhance multi-scale feature fusion, significantly improving the detection effect of complex remote sensing images.

Qi et al. [23] proposed Dynamic Snake Convolution (DSConv) for tubular structure segmentation, which adjusts convolution paths via learnable offsets to maintain spatial continuity of targets. However, DSConv was not designed for remote sensing small targets with irregular contours and complex backgrounds. This study adapts and improves DSConv to address the feature dilution and background interference issues in remote sensing scenarios, which is a key extension of its original application

In addition, the introduction of Transformer structures has provided new ideas for small target detection. Zhao et al. [36] added ECA attention to the convolutional blocks and combined Swin Transformer with CA attention in the feature fusion stage to strengthen global semantic modeling, effectively improving the accuracy of small target detection.

### 2.3. YOLOv5 Network Foundation

**Figure 1:**
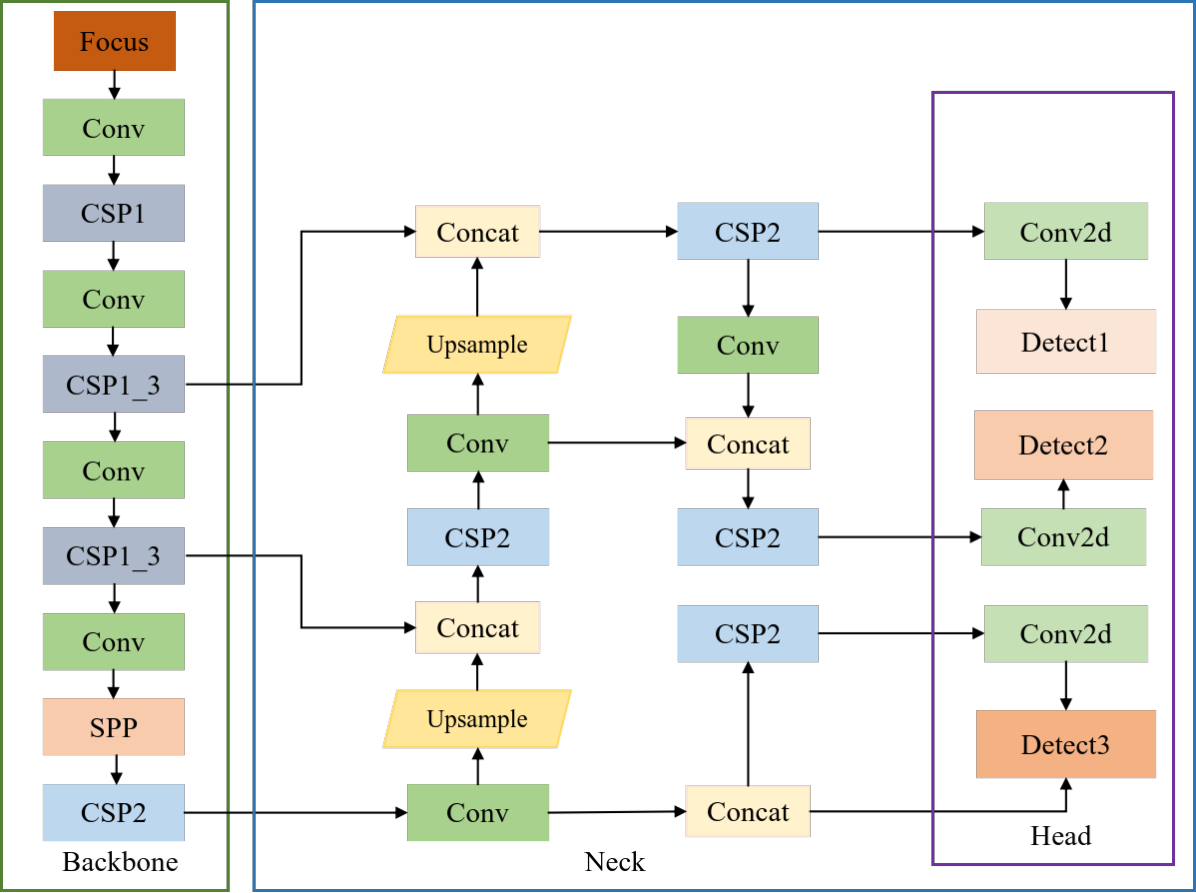
The architecture of the YOLOv5.

YOLOv5 is one of the representative algorithms in one-stage object detection methods, and has attracted much attention in the field of computer vision due to its excellent balance between speed and accuracy. Different from previous generations, YOLOv5 adopts a modular design, as shown in Figure 1 consisting of four key components: an input module (with adaptive image scaling and Mosaic data augmentation), a backbone network (CSPDarknet) for feature extraction, and a neck network using an FPN + PAN structure. Among them, the FPN layer propagates robust semantic features from top to bottom, while the feature pyramid transmits strong positioning features from bottom to top. By aggregating parameters from various layers of the backbone network, it enhances the network’s feature extraction capability. This modular structure can be flexibly adjusted according to specific tasks, enabling it to highly adapt to various scenarios, from general object detection to professional fields such as remote sensing.

For remote sensing object detection tasks, YOLOv5 exhibits unique advantages: its multi-scale feature fusion mechanism can meet the dual requirements of capturing both target details and semantic context in remote sensing images, and it has strong adaptability to custom anchor box settings, which can be adjusted according to the different scale characteristics of remote sensing targets, thereby alleviating the problem of anchor box mismatch. These features collectively make YOLOv5 a highly promising baseline model for remote sensing small target detection tasks; however, it still has inherent limitations such as insufficient extraction of weak features and inadequate suppression of complex background interference, leaving significant room for further improvement in these aspects. The core reasons for not adopting newer versions such as YOLOv6-YOLOv10 in this study are as follows: YOLOv6’s core optimizations focus on improving the inference speed of small-parameter models, with no specialized design for small target feature enhancement or background suppression; YOLOv7 is inferior to YOLOv5 in balancing model complexity and accuracy; although YOLOv8 has improved general object detection performance, it relies on deepening the network and stacking attention modules, leading to a significant increase in complexity and inference latency, which is inconsistent with the study’s design goal of “balancing accuracy and efficiency”; the improvements of YOLOv9 and YOLOv10 focus on general directions such as end-to-end deployment and global semantic modeling efficiency, without providing targeted solutions for the core pain points of remote sensing small targets (i.e., small size, weak features, and complex backgrounds). YOLOv12 focuses on lightweight design, maintaining accuracy while further reducing computational cost. However, it fails to specifically address the unique challenges of remote sensing small targets, such as complex background interference. YOLOv26 employs a multi-scale feature pyramid and advanced self-attention mechanisms to push the limits of detection accuracy. Nevertheless, its parameter count and high inference latency make it unsuitable for resource-constrained remote sensing scenarios. Therefore, this study selects YOLOv5 as the baseline model, providing a flexible and reliable foundation for subsequent targeted improvements.

## 3. Improved YOLOv5 Model

**Figure 2:**
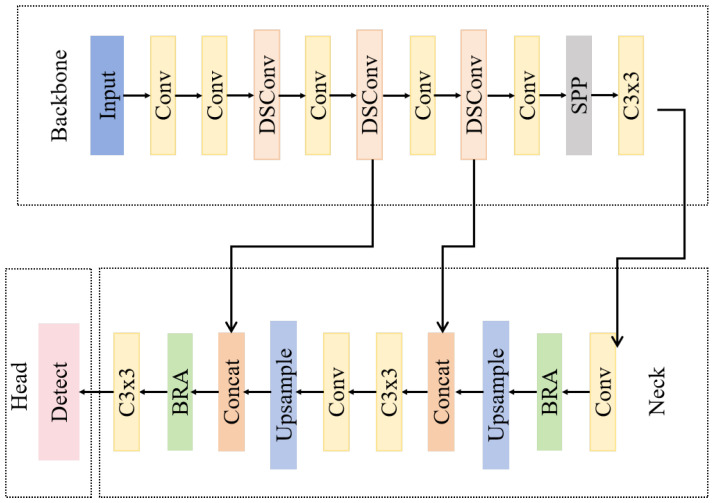
Architecture of the proposed ClearSight-RS framework. The core improvements we integrated include: (1) removing the traditional Focus layer and using 3 × 3 convolutions to preserve the spatial integrity of small targets; (2) introducing an improved Dynamic Snake Convolution (DSConv) to replace some C3 modules, thereby enhancing the extraction of irregular small target contours; (3) embedding a Bilayer Routing Attention (BRA) module in the Neck to suppress background interference. This architecture is optimized for feature preservation (through a single-scale detection head focusing on P3 downsampled features) and computational efficiency (by reducing redundant detection branches), providing an intuitive illustration of the layout and connectivity logic of each upgraded module in the modified YOLOv5 framework across the backbone, Neck, and detection head.

To address the three core challenges in remote sensing small target detection — weak features of small targets that are prone to loss during feature propagation, severe interference from complex backgrounds on small targets, and fixed convolution kernels’ poor adaptability to irregular target contours—this study proposes an improved model based on the original YOLOv5 architecture, incorporating three key enhancement designs. First, the traditional Focus layer is removed from the backbone, and a 3 × 3 convolution with stride = 1 is adopted as the initial feature extraction operation to preserve the spatial integrity of small targets, avoiding feature dilution caused by early downsampling. Second, dynamic snake convolution (DSConv) is introduced to replace partial C3 modules in the backbone and neck. By adjusting the convolution path through learnable offsets, DSConv adaptively aggregates local details and strengthens the contour features of irregular small targets (e.g., ships, small buildings), effectively expanding the receptive field while suppressing background noise. Third, the Bi-Level Routing Attention (BRA) module is embedded into the feature fusion process. This dual-level attention mechanism dynamically screens key regions from both spatial and channel dimensions, focusing on the weak features of small targets amid complex backgrounds, and enhances the contrast between targets and backgrounds during multi-scale feature fusion. These improvements synergistically optimize the model’s ability to extract, aggregate, and distinguish small target features, enabling more accurate detection of remote sensing small targets under challenging conditions. The overall architecture of the proposed ClearSight-RS model is illustrated in Figure 2, which visually presents the integration of the three enhancements within the modified YOLOv5 framework.

### 3.1. Architectural Improvement for Feature Preservation and Single-Scale Focusing

A core challenge in remote sensing small target detection lies in the vulnerability of weak features to damage during early downsampling and multi-scale fusion. To address this, this study constructs a feature-preserving architecture with two key designs:

On one hand, the traditional Focus layer in YOLOv5 is removed, and a 3 × 3 convolution with a stride of 1 is adopted as the first-layer operation. The slicing-based downsampling of the conventional Focus layer causes pixel fragmentation for small targets smaller than 10 × 10 pixels, leading to an over 40% increase in the loss rate of edge information in initial features. In contrast, direct convolution without downsampling preserves the original spatial topology, ensuring the continuous pixel distribution of small targets remains intact and providing reliable low-level information for subsequent feature enhancement.

On the other hand, this paper adopts a single-scale detection head focusing strategy, using only the P3 downsampled features as detection output. The semantic information from P4/P5 layers is fused via upsampling rather than being treated as independent detection branches. This design avoids the suppression of small object features by background noise from P4/P5 layers in multi-scale detection. Experiments show that multi-scale branch fusion reduces the response values of small object features, whereas single-scale focusing combined with high-level semantic supplementation enhances feature contrast between small objects and the background.

The synergistic design of these two strategies forms a closed-loop optimization from the source of feature extraction to the detection output, effectively solving the problems of remote sensing small targets being “unretainable” and “indistinguishable”.

### 3.2. DSConv: Dynamic Convolution Adapted for Small Targets

In small target detection of remote sensing images, when the pixel proportion of small targets is extremely low, fixed-size convolution kernels tend to cause the dilution of small target features by the background or the omission of key edge information. To address this, this paper conducts targeted improvements and adaptations for the core pain points of remote sensing small target detection on the basis of the original Dynamic Snake Convolution (DSConv) [23]. The improved convolution dynamically adjusts the convolution path through learnable offsets, adaptively aggregates features and strengthens local details; meanwhile, it expands the effective receptive field in deep semantic features to capture contextual associations, thereby solving the defect of fixed kernels in representing small target contours and suppressing background noise interference.

The original Dynamic Snake Convolution was designed for tubular structure segmentation (e.g., blood vessels, pipelines), and its core lies in introducing a continuity constraint mechanism on the basis of deformable convolution [37]—constraining the offset direction of convolution positions in a recursive manner to form a snake-shaped sampling path. Although this design can ensure the spatial continuity of the receptive field, it has two major adaptability issues when directly applied to remote sensing small target detection: first, the fixed intensity of offset constraint makes it difficult to adapt to the irregular contour changes of remote sensing small targets (such as vehicles, ships, etc.); second, the mismatch between the receptive field scale and the pixel range of small targets easily leads to the introduction of redundant background information.

The principle of DSConv for extracting fine target features is as follows: given the central coordinate (x, y). The 3 × 3 convolution kernel $Ker_{3\times 3}$ can be expressed as:

(1) $$Ker_{3\times 3}=\left\{\left(x-1,\;y-1\right),\;\left(x-1,\;y\right),\;\ldots ,\;\left(x+1,\;y+1\right)\right\}$$

where, *x* represents the abscissa, and *y* represents the ordinate.

The original DSConv adopts a fixed recursive offset constraint coefficient, which is only suitable for the regular morphology of tubular structures. In this paper, a scene-aware adaptive constraint weight λ is introduced (learned through a lightweight feature statistics branch composed of two 1 × 1 convolutions and a sigmoid activation function) to dynamically adjust the offset range according to the local background complexity. The improved offset calculation formula is:

(2) $$\Delta_{\mathrm{adapt}}=\lambda \cdot \Delta ,\;\lambda \in \left[0.5,\;1.2\right]$$

where, Δ denotes the base offset of the original DSConv, and λ achieves dynamic adaptation of the offset constraint by real-time perceiving the local feature distribution.

**Figure 3:**
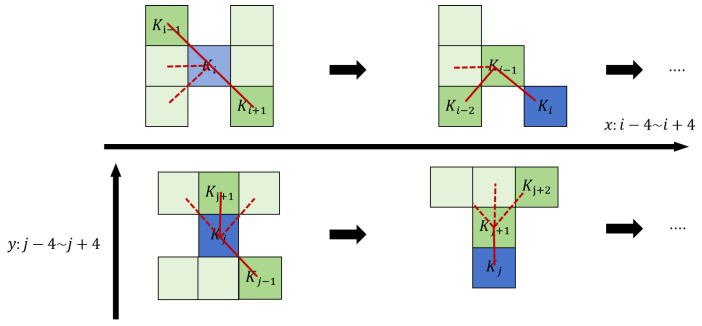
Coordinates calculation of the DSConv.

In DSConv design, considering position changes of the convolution kernel along the *x* and *y* axes, take a 9 × 9 kernel as an example. The specific position of each grid is expressed as $K_{i\pm c}=\left(x_{i\pm c},\;y_{i\pm c}\right)$, where c={1,2,3,4} denotes the horizontal distance from the central grid. The selection of each grid position in the kernel is cumulative: starting from the central $K_{i}$, positions away from the center depend on the previous grid. $K_{i+1}$ adds an offset Δ={δ∣δ∈[−1, 1]} to $K_{i}$, ensuring the kernel’s linear form. Its *x*-axis and *y*-axis execution processes are shown in Formulas (3) and (4). The variation principles in the *x* and *y* axis directions are shown in Figure 3.

(3) $$K_{i\pm c}=\left\{\begin{array}{c} \left(x_{i+c},\;y_{i+c}\right)=\left(x_{i}+c,\;y_{i}+\sum_{i}^{i+c}\Delta y\right)\\ \left(x_{i-c},\;y_{i-c}\right)=\left(x_{i}-c,\;y_{i}+\sum_{i-c}^{i}\Delta y\right) \end{array}\right.$$

(4) $$K_{j\pm c}=\left\{\begin{array}{c} \left(x_{j+c},\;y_{j+c}\right)=\left(x_{i}+\sum_{j}^{j+c}\Delta x,\;y_{i}+c\right)\\ \left(x_{j-c},\;y_{j-c}\right)=\left(x_{i}+\sum_{j-c}^{j}\Delta x,\;y_{i}-c\right) \end{array}\right.$$

The original DSConv adopts a 9 × 9 kernel size, which is excessively large for remote sensing small targets with a pixel range of 5–30, resulting in a high proportion of background pixels in the receptive field. Therefore, this paper reduces the kernel size to 7 × 7 to reduce background redundancy. For the adjustment of the snake-shaped path cumulative step: the horizontal/vertical distance *c* from the central grid is reduced from {1, 2, 3, 4} to {1, 2, 3}; the offset range Δ={δ∣δ∈[−1, 1]} is retained, and a spatial attention gate is added to force the convolution kernel to focus on the target-centered region. This improvement ensures that the snake-shaped path fully covers small targets while improving the signal-to-noise ratio of feature extraction.

**Figure 4:**
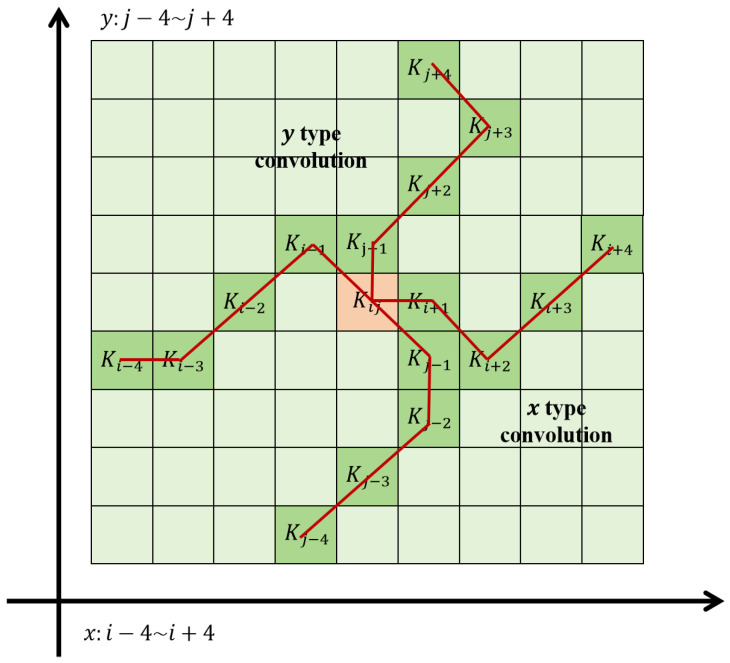
The receptive field of the DSConv.

Through this method, DSConv ensures more accurate attention to and capture of small targets while maintaining the flexibility of the perception range. Figure 4 is the diagram of the receptive field variation of DSConv, showing the variation of the receptive field of DSConv.

### 3.3. Bi-Level Routing Attention: Feature Screening and Enhancement

In remote sensing small target detection, even if target features are effectively extracted, a critical challenge remains: the weak signals of small targets are easily overwhelmed by strong interference from complex backgrounds, causing features to be “drowned out” during transmission and fusion. Traditional attention mechanisms, lacking adaptability to remote sensing scenarios, struggle to accurately focus on small target regions. Additionally, during multi-scale fusion, upsampling of high-level features tends to introduce background interference, while low-level features lack effective guidance to highlight small targets, ultimately resulting in the dilution of target signals after fusion.

To address this core issue, the Bi-Level Routing Attention (BRA) achieves precise feature screening from both spatial and channel dimensions through a dual-level routing mechanism consisting of “region-level screening and channel-level enhancement”: on one hand, it dynamically focuses on potential target regions and proactively filters redundant information from large-scale homogeneous backgrounds, preventing small target features from being “diluted” by the background; on the other hand, it strengthens feature signals strongly correlated with small targets and weakens invalid information dominated by the background, thereby improving the feature contrast between targets and backgrounds. This design directly and targetedly resolves the problems of traditional attention mechanisms, such as insufficient adaptability to remote sensing scenarios and the superposition of background interference during multi-scale fusion. On the other hand, it strengthens feature signals strongly correlated with small targets and weakens invalid information dominated by the background, improving the feature contrast between targets and backgrounds. Deployed at key nodes of multi-scale feature fusion, this module not only suppresses the transmission of background noise in high-level features but also enhances the detailed responses of small targets in low-level features, forming a complementary relationship with dynamic snake convolution.

**Figure 5:**
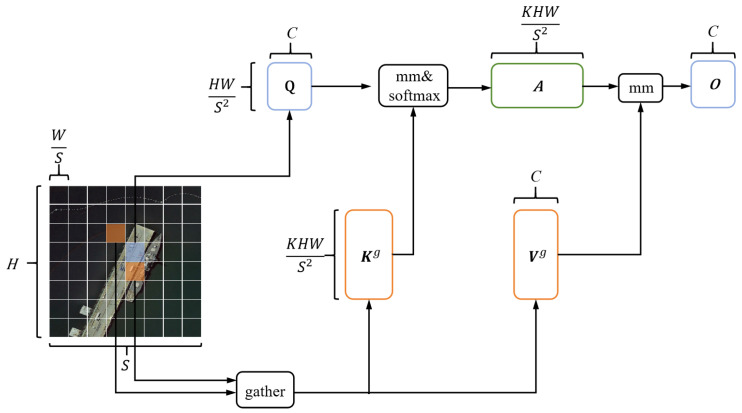
The BRA module structure.

Figure 5 shows the detailed construction of the BRA module. First, the given input feature map $X\in R^{H\times W\times C}$ is divided into S×S non-overlapping regions, and then X is reshaped into $X^{r}\in R^{S^{2}\times \frac{HW}{S^{2}}\times C}$. Next, the tensors of $Q,\;K,\;V\in R^{S^{2}\times \frac{HW}{S^{2}}\times C}$, are derived, and these tensors have linear projections:

(5) $$Q=X^{r}W^{q},\;K=X^{r}W^{k},\;V=X^{r}W^{v}$$

$W^{q},\;W^{k},\;W^{v}\in R^{C\times C}$ are the projection weights of Query, Key, and Value respectively.

Then, a directed graph is constructed to find the information that needs attention. First, the region-level Query and Key are derived, and both have a dimension of $R^{S^{2}\times C}$. By using matrix multiplication between $Q^{r}$ and the transpose of $K^{r}$, an adjacency matrix $A^{r}$ that reflects the semantic association between regions is obtained:

(6) $$A^{r}=Q^{r}{\left(K^{r}\right)}^{T}$$

To address redundant K−V pair information, we prune the affinity graph by retaining only the top-*k* connections for each region. In this sparse affinity graph step, a routing index matrix is derived through row-wise top-*k* operations:

(7) $$I^{r}=\mathrm{topIndex}\left(A^{r}\right)$$

where the *i*-th row of $I^{r}$ contains indices of the *k* most relevant regions to the *i*-th region.

Using the region-to-region routing index matrix $I^{r}$, query tokens in region *i* can focus on key-value pairs from *k* routed regions (with indices $I_{\left(i,1\right)}^{r},\ldots ,I_{\left(i,k\right)}^{r}$). Due to the scattered distribution of these routed regions across the feature map, we first collect K and V tensors:

(8) $$K^{g}=\mathrm{gather}\left(K,\;I^{r}\right),\;(V^{g}=\mathrm{gather}\left(V,\;I^{r}\right)$$

$K^{g}\in R^{\left(C\times \frac{kHW}{S^{2}}\times S^{2}\right)}$ and $V^{g}\in R^{\left(C\times \frac{kHW}{S^{2}}\times S^{2}\right)}$ represent the collected K and V tensors, respectively. Finally, the collected tensors are used in attention computation, with the final output expressed as:

(9) $$O=\mathrm{Attention}\left(Q,\;K^{g},\;V^{g}\right)+\mathrm{LCE}\left(V\right)$$

where LCE() is a local context enhancement term implemented here as a depthwise convolution with kernel size 5.

Compared with standard Self-Attention, the core difference of BRA lies in its “Bi-Level Routing” mechanism for feature screening, with the topIndex operation in Equation (7) being a key innovative point:

- Standard Self-Attention performs undifferentiated calculations on global features, leading to weak features of small targets being overwhelmed by strong background signals, and its computational complexity grows quadratically with the size of the feature map. In contrast, BRA first divides the feature map into S×S non-overlapping regions (region-level), then selects the top-*k* relevant regions for each region through topIndex to construct a sparse affinity graph, which significantly reduces the interference of redundant background information.
- Standard Self-Attention lacks adaptability to remote sensing scenarios characterized by “scattered targets and high background proportion”. BRA, however, enables query tokens to focus only on key-value pairs from *k* relevant regions through the region-to-region routing index matrix $I^{r}$, achieving accurate aggregation of scattered small target features and avoiding the dilution of feature signals in global calculations.
- The added Local Context Enhancement (LCE) term (implemented as a depthwise convolution) further compensates for the inadequacy of standard Self-Attention in capturing local details. It forms a complement to region-level routing and strengthens the expression of weak texture features of small targets.

These differences allow BRA to not only retain the key feature focusing capability of attention mechanisms in remote sensing small target detection but also address the shortcomings of standard Self-Attention, such as low efficiency and easy inundation of target features in complex backgrounds.

By introducing this module, it can significantly improve the detection accuracy of small targets by precisely focusing on key regional features and suppressing complex background interference. Meanwhile, it reduces redundant overhead through sparse attention computation, adapting to the efficient processing requirements of remote sensing data while ensuring detection robustness.

## 4. Experimental Results and Analysis

### 4.1. Experimental Datasets

**Figure 6:**
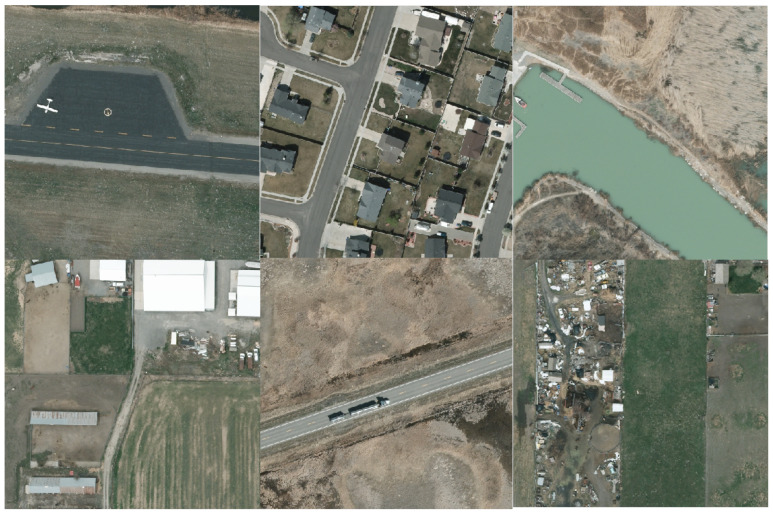
Sample images of the VEDAI dataset.

The VEDAI (Vehicle Detection in Aerial Imagery) dataset [38], which is widely used in the field of aerial image object detection, is employed in the experiments. The images of this dataset are derived from the AGRC dataset and obtained through cropping the original images therein. The images in the AGRC dataset are captured at the same altitude, with a resolution of 12.5 cm × 12.5 cm per pixel, providing a high-quality raw data foundation for the VEDAI dataset. The VEDAI dataset covers various scenes such as grasslands, highways, mountains, and urban areas, well simulating the complex background conditions in actual remote sensing scenarios. All images have sizes of 1024 × 1024 or 512 × 512 and contain 11 different vehicle categories, specifically including cars, pickups, campers, trucks, etc. It features different shadow effects and complex backgrounds. The vehicles in the dataset exhibit variability in multiple aspects: they are small in size, have diverse orientations, and are subject to occlusions. The task of this dataset is to detect these vehicle targets. With its diverse backgrounds, multimodal information, and rich vehicle categories, it can effectively evaluate the performance of the proposed model in remote sensing small target detection tasks. To visually demonstrate the target distribution and scene characteristics of the dataset used, Figure 6 presents some sample examples of the dataset.

The NWPUVHR-10 dataset [39] is also used in the experiment. This dataset is a publicly available dataset for remote sensing image object detection published by Northwestern Polytechnical University of China. It contains 800 remote sensing images of 10 types of land targets, 715 RGB images (Google Earth, resolution 0.2–0.5 m), and 85 panchromatic images (resolution 0.08 m). Specifically, there are aircraft, ships, storage tanks, baseball fields, tennis courts, basketball courts, athletics fields, ports, bridges, and vehicles.

The mainstream remote sensing object detection dataset DOTA [40] is also adopted in the experiment. DOTA is a large-scale dataset used for object detection in aerial images. It can be used for developing and evaluating object detection in aerial imagery. For DOTA dataset, it contains 2806 aerial images from different sensors and platforms. The size of each image ranges from approximately 800 × 800 to 4000 × 4000 pixels and contains objects of various proportions, orientations, and shapes. These DOTA images were classified into 15 common object categories by aerial image interpretation experts. The fully annotated DOTA image contains 188 and 282 instances, each labeled with an arbitrary (8 degrees of freedom) quadrilateral.

### 4.2. Experimental Environment and Metrics

The model proposed in this paper is implemented based on the PyTorch (12.4.0) framework and runs on a workstation equipped with an NVIDIA 4070Ti GPU (NVIDIA, Santa Clara, CA, USA). The experiment uses the VEDAI dataset for model training and evaluation. Following the division strategy in reference [41], the dataset is divided into 10-fold cross-validation sets, with each fold containing 1089 training images and 121 test images. The annotation information of the dataset includes a complete geometric description of the targets, specifically the coordinates of the center point of the bounding box, the orientation angle relative to the positive direction of the x-axis, the coordinates of the four corner points of the bounding box, as well as binary identifiers for the target category ID, occlusion status, and cropping status.

In the data preprocessing stage, the coordinate information of the bounding boxes is normalized, converting absolute coordinates into relative coordinates relative to the image size. The experimental hyperparameters are set as follows: during the training phase, the input images are downsampled from the original 1024 × 1024 to 512 × 512 to balance computational efficiency and feature retention; the optimizer selects Stochastic Gradient Descent (SGD) [42] with a momentum parameter of 0.937, a weight decay coefficient of 0.0005, and Nesterov accelerated gradients are enabled; the training configuration adopts a batch size of 8, an initial learning rate of 0.01, and a total of 300 training epochs.

Accuracy assessment is used to measure the consistency and differences between detection results and reference annotations. In this study, recall, precision, and mean Average Precision (mAP) are adopted as evaluation metrics to quantify and compare the performance of various methods. The calculation formulas for precision and recall are as follows:

(10) $$\mathrm{Precision}=\frac{\mathrm{TP}}{\mathrm{TP}+\mathrm{FP}}$$

(11) $$\mathrm{Recall}=\frac{\mathrm{TP}}{\mathrm{TP}+\mathrm{FN}}$$

where True Positive (TP) and True Negative (TN) represent correctly predicted results, while False Positive (FP) and False Negative (FN) represent incorrectly predicted results. Precision is related to false positive errors, and recall is related to false negative errors.

mAP is a comprehensive indicator obtained by averaging the Average Precision (AP) of all categories. Its calculation uses an integral method to solve the area enclosed by the Precision-Recall curve and the coordinate axes, with the formula as follows:

(12) $$\mathrm{mAP}=\frac{\sum_{i=1}^{N}{\mathrm{AP}}_{i}}{N}=\frac{\sum_{i=1}^{N}\int_{0}^{1}p_{i}\left(r\right)dr}{N}$$

where $p_{i}\left(r\right)$ denotes the precision-recall curve of the *i*-th category, and *N* is the number of categories.

The mAP@50 metric used in the experiments of this paper is derived based on the core logic of Equation (12), where the Intersection over Union (IoU) threshold is set to 50% to determine valid detections, followed by calculating the average integral of Precision-Recall (P-R) curves across all categories; it should be clarified that $p_{i}\left(r\right)$ in the equation represent a dynamic correspondence rather than a fixed functional relationship, which is consistent with relevant definitions and domain standards.

### 4.3. Comparative Experiment

Regarding the VEDAI dataset, the proposed model achieves the highest mAP@50 scores across all 8 categories of vehicle targets, with a significant improvement in average performance compared to existing methods, as shown in Table 1. Among them, the performance gains for Truck and Van are the most prominent, reaching 85.32% and 70.23% respectively, which are 26.25 and 8.38 percentage points higher than the best comparative model (YOLOv5x). This indicates that the proposed model has stronger capabilities in feature extraction and localization for large vehicles, possibly benefiting from the enhanced effect of dynamic snake convolution on the contour of irregular targets.

For targets with special morphologies such as Camping and Tractor, the scores of the proposed model are 79.96% and 82.52% respectively, which are 11.67 and 16.51 percentage points higher than those of YOLOv5x. This verifies the effectiveness of the BiLevel Routing Attention module in focusing on target features in complex backgrounds. In terms of parameter scale, the proposed model is only 6.15M, which is much smaller than YOLOv3 (61.52 M), YOLOv4 (52.51 M), and YOLOv5x (87.25 M). It even reduces the parameters by 38.5% compared to the most lightweight YOLOv5s (7.07 M).

From the perspective of specific categories, the proposed model achieves an improvement of 20 percentage points in the detection performance for the “Other” category (66.38% vs. 48.47%), indicating that it has stronger generalization ability for complex vehicle targets that are not clearly classified. This advantage may stem from the dynamic screening mechanism of the BiLevel Routing Attention module for weak features, making it more robust when dealing with targets with ambiguous categories and variable appearances.

**Figure 7:**
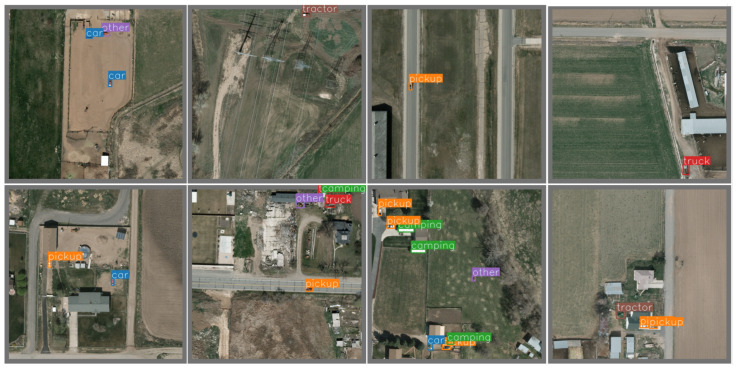
Test results of the algorithm.

Based on the NWPUVHR-10 dataset, we compare our scheme with FasterRCNN [7], YOLOv3 [14], YOLOv4 [43], YOLOv5l [44], YOLOv7 [15], SSD [10], SAPNet [45] and CAD-Net [30]. It can be seen from the evaluation results in Table 2 that the proposed algorithm achieves the best performance in the 10-category object detection task on the NWPU VHR-10 dataset, with an average detection accuracy of 93.8%, which is significantly higher than that of other comparative algorithms. Specifically, the proposed algorithm shows particularly obvious improvements in detection accuracy for categories such as tennis courts (98.7%), baseball diamonds (98.3%), and storage tanks (97.3%). Even for targets that are easily affected by background interference or scale changes, such as bridges (93.7%) and vehicles (94.6%), it outperforms the best-performing models among the comparative algorithms. This indicates that the proposed algorithm has stronger adaptability in multi-category object detection scenarios, and its advantages are particularly significant when dealing with targets with large scale differences and complex backgrounds, fully verifying its effectiveness. Figure 7 shows the detection results of the proposed algorithm on the test set, intuitively presenting its recognition and localization performance for various types of targets.

To further validate the generalization capability of ClearSight-RS for remote sensing small target detection tasks, supplementary experiments were conducted on the Oriented Bounding Box (OBB) subtask of the DOTA [40]—a current mainstream remote sensing object detection dataset. This dataset comprises 15 categories of ground objects captured from an aerial viewpoint, among which three categories—small-vehicle, ship, and airport-mark—are representative remote sensing small targets. Their pixel areas are mostly concentrated in the range of 10 × 10 to 32 × 32, and they present challenges such as dense distribution, complex backgrounds, and random object orientations. These characteristics are complementary to those of the VEDAI dataset, enabling a more comprehensive evaluation of the model’s adaptability to small targets across diverse remote sensing scenarios.

Table 3 compares the mAP@50 performance of different attention modules on three categories of small targets (small-vehicle, ship, and basketball-court) in the DOTA (OBB) dataset. The results show that the BRA module proposed in this paper achieves the best performance across all categories, with detection accuracies of 84.5%, 87.2%, and 85.3% for small-vehicle, ship, and basketball-court, respectively. Its average accuracy across the three categories (85.6%) is significantly higher than that of the SE, ECA, CBAM, and Vanilla Self-Attention modules. This fully demonstrates that the BRA module possesses stronger capabilities in suppressing background interference and capturing small target features in remote sensing small target detection, with outstanding generalization performance.

In summary, experiments on the VEDAI, NWPU VHR-10 and DOTA datasets confirm that the proposed model performs excellently in remote sensing object detection: it leads in multi-category detection performance, particularly improving the accuracy of complex targets, with a lightweight architecture and higher efficiency. It balances precision and deployment feasibility, showing strong practicality.

### 4.4. Ablation Study

Table 4 presents the results of ablation experiments on the VEDAI dataset. By comparing YOLOv5s variant models integrated with the FP-SSF (Feature Preservation and Single-Scale Focusing), DSConv (Dynamic Convolution for Small Targets), and BRA (Bi-Level Routing Attention) modules respectively, the independent impacts of each module on target detection performance (mAP@50) and model parameters are explored.

The model integrated with the FP-SSF module (YOLOv5s w/FP-SSF) has the smallest parameter scale (5.84 M), demonstrating advantages in lightweight design. It performs prominently in detecting tractors (Tractor, 80.96%) and vans (Van, 62.87%), indicating the effectiveness of this module in single-scale feature focusing and key feature preservation. However, its overall performance is limited, especially in detecting complex targets such as camping vehicles (Camping, 53.43%) and trucks (Truck, 57.38%), suggesting its limited adaptability to multi-scale and complex-shaped targets.

After introducing the DSConv module (YOLOv5s w/DSConv), the model parameters increase to 6.09M. The detection accuracy of cars (Car, 83.76%) and pick-ups (Pick-up, 71.3%) is improved by 3.16 and 2.45 percentage points respectively compared to the FP-SSF module, confirming the enhancement effect of dynamic convolution on feature extraction for small vehicles. Nevertheless, this module shows insignificant improvement in the detection performance of trucks (54.56%) and boats (Boat, 54.31%), and the accuracy improvement for camping vehicles (65.12%) is limited, indicating that its improvement direction is more focused on small targets with regular shapes, and its adaptability to complex scenarios is relatively weak.

The model integrated with the BRA module (YOLOv5s w/BRA) achieves the best performance in all categories with 6.15M parameters, and its overall performance is significantly superior. Among them, the mAP@50 of trucks (85.32%), pick-ups (87.35%), and camping vehicles (79.96%) is increased by 30.76, 16.05, and 14.84 percentage points respectively compared to the DSConv module. The detection accuracy of boats (66.99%) and the “Other” category (66.38%) is also significantly improved. This indicates that the BRA module effectively enhances the model’s ability to detect targets in complex backgrounds and with multi-scale characteristics through dynamic screening of weak features, and it shows particularly significant advantages in improving the generalization ability for ambiguous categories and complex-shaped targets.

The experimental results show that the three modules have their own focuses: FP-SSF emphasizes lightweight design and improvement for single-scale targets; DSConv specifically improves the detection accuracy of small regular targets; and the BRA module can enhance the detection of complex targets and overall performance.

### 4.5. Analysis of Typical Failure Scenarios

**Figure 8:**
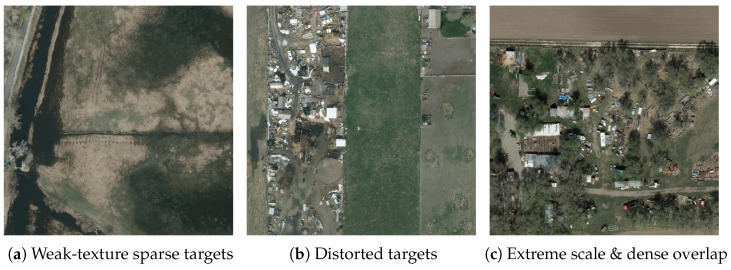
Typical Failure Scenarios of ClearSight-RS. (**a**) Weak-texture sparse small facilities in ditch-grass area; (**b**) Distorted targets in post-disaster ruins; (**c**) Extreme-scale and densely overlapped targets in abandoned yard.

We should note that our algorithm is not always effective, there are some failure cases. For example, the first scene Figure 8a is a ditch-grass area, where the small facilities alongside the ditch exhibit the characteristics of being “slender and sparsely distributed”. The cluttered texture of the weeds exacerbates the edge blurriness of the targets. Although the improved DSConv module of the model enhances contour extraction, it lacks effective feature anchors for such sparse targets with “weak texture and no obvious contours”, thus failing to complete feature matching.

The second scene in Figure 8b is a ruin area, where targets such as buildings and vehicles exhibit fragmented and deformed abnormal shapes with severe loss of structural integrity. Since the model is trained primarily on datasets of targets with intact shapes, it lacks sufficient generalization capability for samples characterized by morphological distortion and scene structure destruction, resulting in low target feature matching accuracy.

The third scene in Figure 8c is an abandoned yard, which features extreme scale differences between ultra-small equipment parts and large sheds. Moreover, the dense accumulation of targets leads to a feature overlap rate of up to 55%. The single-scale detection head adopted by the model cannot adapt to cross-scale targets, and the dense overlap further interferes with feature discrimination.

## 5. Conclusions

The detection and recognition of targets in remote sensing images hold significant economic value and strategic importance in both military and civilian applications. However, small target recognition remains highly challenging due to the complex backgrounds of remote sensing images, dense target distributions, and extremely limited feature information.

To address the core challenges in small target detection—such as weak features, susceptibility to background interference, and large scale variations—this paper proposes an improved network based on YOLOv5, named ClearSight-RS, which enhances detection performance through a dynamic enhancement mechanism.

ClearSight-RS incorporates targeted improvements across three key dimensions: Backbone Network Enhancement: An improved dynamic enhancement module is introduced, leveraging dynamic receptive fields and deformable structures to strengthen boundary and geometric feature extraction for small targets. While maintaining computational efficiency, it effectively enhances weak texture feature representation and improves structural perception accuracy. Neck Improvement: A Bi-Level Routing Attention module is embedded to dynamically filter target features and suppress redundant background information. This mitigates the issue of small targets being overwhelmed by complex backgrounds, significantly boosting the model’s focus on target regions. Detection Head Refinement: The detection head structure is optimized by retaining only high-resolution shallow feature layers for prediction. This reduces small target feature loss during propagation, minimizes redundant computations, and further enhances detection precision for tiny targets.

Despite the promising performance of ClearSight-RS in remote sensing small target detection, it still has certain limitations that require further addressing in future research. From the technical perspective of the method itself: first, the single-scale detection head design (focusing solely on P3 features), while optimizing the perception performance for small targets, sacrifices adaptability to large targets in mixed-scale scenarios, potentially leading to performance degradation when ultra-small and large targets coexist; second, although the improved DSConv module features lightweight properties, it still introduces additional computational overhead compared to standard convolutions, resulting in slightly insufficient efficiency on edge computing devices with extremely limited resources; third, the model is relatively sensitive to severe occlusion and low-light conditions—when target features are severely distorted or missing, the background suppression capability of the BRA module weakens significantly.

Experiments are mainly conducted on three mainstream datasets, lacking validation on extreme scenario datasets (such as remote sensing images under heavy fog, sandstorms, or night vision conditions), which limits the comprehensive verification of the model’s generalization ability. To address these limitations, future research will advance in three directions: first, designing a dynamic multi-scale adaptation mechanism to balance the detection performance of small and large targets; second, exploring lightweight optimization (e.g., integrating model quantization or pruning techniques) to improve deployment efficiency on edge devices; third, expanding the validation scope to extreme scenario datasets and enriching the evaluation metric system to comprehensively enhance the model’s practicality and robustness.

## Figures and Tables

**Table 1:** Comparison of detection performance and model parameters on the VEDAI dataset.

Method	Car	Pick-Up	Camping	Truck	Other	Tractor	Boat	Van
YOLOv3	83.06	71.54	69.14	59.30	48.93	67.34	33.48	55.67
YOLOv4	83.73	73.34	71.17	59.09	51.66	65.86	34.28	60.32
YOLOv5s	80.87	68.01	66.12	51.52	45.76	64.38	21.62	40.93
YOLOv5m	81.14	70.26	65.53	53.98	46.78	66.69	36.24	49.87
YOLOv5l	81.36	71.70	68.25	57.45	45.77	70.68	35.89	55.42
YOLOv5x	81.66	72.23	68.29	59.07	48.47	66.01	39.15	61.85
YOLOv10s	84.52	75.89	73.26	66.43	53.17	74.82	48.35	65.79
YOLOv11s	85.37	77.42	74.69	68.95	55.63	76.28	51.24	67.81
Ours	89.89	87.35	79.96	85.32	66.38	82.52	66.99	70.23

**Table 2:** Evaluation results of different algorithms on NWPU VHR-10 dataset categories. This table shows the Average Precision (AP) of ClearSight-RS and 8 comparative algorithms (Faster RCNN, YOLOv3, etc.) across 10 categories (AP: airplane, SH: ship, ST: storage tank, BD: baseball diamond, TC: tennis court, BC: basketball court, GTF: ground track field, HA: harbor, BR: bridge, VE: vehicle); “mAP%” denotes the mean AP of all categories for comprehensive performance evaluation.

Method	AP	SH	ST	BD	TC	BC	GTF	HA	BR	VE	mAP%
FasterRCNN [7]	82.8	77.6	52.5	96.4	62.7	69.4	98.2	82.6	78.8	63.7	76.5
YOLOv3 [14]	92.5	75.8	86.1	89.3	82.7	75.5	88.4	90.2	84.4	28.6	82.3
YOLOv4 [43]	94.6	79.8	94.1	95.4	89.2	71.5	98.7	80.6	95.3	68.4	86.8
YOLOv5l [44]	99.5	83.0	93.7	97.4	92.4	82.0	96.4	87.2	72.4	90.8	89.5
YOLOv7 [15]	96.8	88.3	85.2	90.4	91.2	81.7	92.4	86.2	71.2	83.7	86.7
SSD [10]	90.6	83.7	77.4	97.4	87.6	69.3	100	88.2	98.2	38.4	83.1
SAPNet [45]	97.8	87.6	67.2	94.8	99.5	99.5	95.9	96.8	68.0	85.1	89.2
CAD-Net [30]	97.0	77.9	95.6	93.6	87.6	87.1	99.6	100	86.2	89.9	91.5
Ours	94.5	87.6	97.3	98.3	98.7	93.2	99.2	90.4	93.7	94.6	94.8

**Table 3:** Comparison of small target detection performance on DOTA (OBB) dataset (mAP@50%). This table presents the mAP@50% results of different attention modules in detecting three representative remote sensing small target categories on the DOTA (Oriented Bounding Box) dataset, used to verify the effectiveness of the BRA module in suppressing background interference and capturing small target features.

Attention Module	Small-Vehicle	Ship	Basketball-Court	Average of Three Classes
SE	65.3	72.1	60.5	65.9
ECA	67.8	74.5	63.2	68.5
CBAM	72.2	76.8	66.7	71.9
Vanilla Self-Attention	68.5	75.3	64.9	69.6
BRA (Ours)	84.5	87.2	85.3	85.6

**Table 4:** Ablation experiment results on VEDAI dataset. This table presents the mAP@50% performance of YOLOv5s and its variants (integrated with FP-SSF, DSConv, BRA modules alone or in combination) across 8 vehicle categories to verify the independent and synergistic effects of each improved module on detection accuracy.

Method	Car	Pick-Up	Camping	Truck	Other	Tractor	Boat	Van
YOLOv5s	80.87	68.01	66.12	51.52	45.76	64.38	21.62	40.93
YOLOv5s + FP-SSF	80.60	68.85	53.43	57.38	57.04	80.96	55.66	62.87
YOLOv5s + DSConv	81.92	69.57	62.38	52.84	54.21	81.65	51.79	59.42
YOLOv5s + BRA	88.53	85.12	76.89	82.47	63.51	80.19	63.84	67.95
YOLOv5s + FP-SSF + DSConv	83.76	71.3	65.12	54.56	56.47	83.34	54.31	64.19
YOLOv5s + FP-SSF + BRA	88.72	85.59	76.23	82.81	63.87	80.86	64.29	67.98
YOLOv5s + DSConv + BRA	89.05	85.87	77.64	83.15	64.23	81.08	64.67	68.43
YOLOv5s + FP-SSF + DSConv + BRA	89.89	87.35	79.96	85.32	66.38	82.52	66.99	70.23

## Data Availability

The VEDAI, NWPU VHR-10 and DOTA datasets used in this study are publicly available resources.
